# Oral Microbiota Perturbations Are Linked to High Risk for Rheumatoid Arthritis

**DOI:** 10.3389/fcimb.2019.00475

**Published:** 2020-01-22

**Authors:** Yanli Tong, Linlin Zheng, Pingying Qing, Hua Zhao, Yanhong Li, Linchong Su, Qiuping Zhang, Yi Zhao, Yubin Luo, Yi Liu

**Affiliations:** ^1^Department of Rheumatology and Immunology, West China Hospital, Sichuan University, Chengdu, China; ^2^Hubei Provincial Key Laboratory of Occurrence and Intervention of Rheumatic Diseases, Enshi, China

**Keywords:** oral microbiome, rheumatoid arthritis, high risk, anti-citrullinated protein autoantibodies, dysbiosis

## Abstract

Oral microbial dysbiosis is known to increase susceptibility of an individual to develop rheumatoid arthritis (RA). Individuals at-risk of RA may undergo different phases of disease progression. In this study, we aim to investigate whether and whereby the oral microbiome communities alter prior to symptoms of RA. Seventy-nine saliva samples were collected from 29 high-risk individuals, who were positive for anti-citrullinated protein antibodies (ACPA) and have no clinical arthritis, 27 RA patients and 23 healthy controls (HCs). The salivary microbiome was examined using 16S ribosomal RNA gene sequencing. Alpha and beta diversity analysis and the linear discriminant analysis were applied to examine the bacterial diversity, community structure and discriminatory taxa between three groups, respectively. The correlation between salivary bacteria and autoantibodies were analyzed. In the “pre-clinical” stages, salivary microbial diversity was significantly reduced comparing to RA patients and HCs. In contrast to HCs, like RA patients, individuals at high-risk for RA showed a reduction in the abundance of genus *Defluviitaleaceae_UCG-011* and the species *Neisseria oralis*, but an expansion of *Prevotella*_6. Unexpectedly, the relative abundance of *Porphyromonas gingivalis*, reported as opportunistic pathogens for RA development, was significantly decreased in high-risk individuals. Additionally, we identified four genera in the saliva from high-risk individuals positively correlated with serum ACPA titers, and the other two genera inversely displayed. In summary, we observed a characteristic compositional change of salivary microbes in individuals at high-risk for RA, suggesting that oral microbiota dysbiosis occurs in the “pre-clinical” stage of RA and are correlated with systemic autoimmune features.

## Introduction

Rheumatoid arthritis (RA) is a systemic autoimmune inflammatory disease that primarily involves the joints. Over the past years, research focusing on the earliest stage of RA has led to the discovery of RA-related systemic inflammation and autoimmunity in the pre-clinical stage. The presence of circulating autoantibodies, elevation of cytokines and chemokines levels, and increase of acute phase reactants precede clinical arthritis (Rantapaa-Dahlqvist et al., 2003; Berglin et al., 2004; Nielen et al., 2004; Jorgensen et al., 2008; Sokolove et al., 2012). Prospective studies define ACPA-positive individuals as populations at risk for developing RA, and the chance in subjects with seropositivity and arthralgia can be as high as nearly 30% within 1 year (van de Stadt et al., 2011). In view of potential preventive strategies, the pre-clinical and earliest stages of RA are likely to represent important therapeutic windows within which disease outcomes can be dramatically modulated. Exploring risk factors and biomarkers for RA, especially in the earliest stage of disease is definitely an urgent need.

Complex interplay between genetic and environmental factors contributes to RA etiopathogenesis. An ancient theory of infectious etiology of RA has now been re-emphasized. Recent studies have further corroborated this theory by indicating mucosal origins of the disease. Data from epidemiological and translational studies suggest that environmental exposure and dysbiosis in mucosal sites (lung, gastrointestinal tract, and oral cavity) have causative roles in the development of RA (Scher et al., 2012, 2013, 2016). Of note, *Porphyromonas gingivalis*, a periodontal pathogen, was capable of producing peptidylarginine deiminase (PAD) enzyme to citrullinated antigens. The presence of antibodies to *P. gingivalis* was positively correlated with the increased titer of ACPA (Mikuls et al., 2012; Lappin et al., 2013). Additionally, a recent study revealed a new species in the oral sites of RA patients which promoted citrullinated antigens production (Konig et al., 2016). This species, *Aggregatibacter actinomycetemcomitans*, led to dysregulated PAD function and release of hypercitrullinated proteins through inducing neutrophil migration and neutrophil extracellular traps (NETs) formation (Hirschfeld et al., 2016; Konig et al., 2016). Animal studies further showed that two periodontal pathogens, *P. gingivalis* and *Prevotella nigrescens* aggravated collagen-induced arthritis in mice (de Aquino et al., 2014). These observations support the theory that oral dysbiosis may be an origin of autoantigen production and a risk factor for RA.

Although the oral microbiome is substantially altered in RA patients (Scher et al., 2012; Zhang et al., 2015), little is known about its status in the initial stages of disease development. Whether this alteration precedes clinically evident arthritis and associated with disease development or merely represents a resultant or concomitant phenomenon of the disease is unclear. As ACPA is highly specific (Schellekens et al., 2000), detectable early and predictive of rapid progression and erosion of RA (Nielen et al., 2004; Ronnelid et al., 2005; Syversen et al., 2008; Rombouts et al., 2015), individuals with ACPA positivity are at increased risk for RA, especially those with arthralgia (van de Stadt et al., 2011). We therefore sought to test whether the oral microbiome exhibits distinct taxonomic features in seropositive individuals at high-risk for RA and provide a potential avenue for early detection, intervention or prevention.

## Materials and Methods

### Participants and Study Design

Individuals at high-risk for RA were recruited from West China Hospital, Sichuan University, China. These subjects had a positive serum antibody for ACPA, with or without arthralgia at the time of enrollment. Absence of arthritis was confirmed by physical examination of 44 joints. RA patients were diagnosed according to the American College of Rheumatology (ACR) 2010 classification for RA. Most RA patients were receiving oral disease-modifying anti-rheumatic drugs (DMARDs) and/or corticosteroids at the time of enrollment. Patients receiving biological agents were excluded. Age, gender and ethnicity-matched healthy controls (HCs) with no personal history of inflammatory arthritis were recruited. ACPA-negative profiles for HCs were obtained from the health management center. Subjects from all three study groups were ≥18 years old. Individuals having a history of antibiotics treatment or surgery in the last 3 months, current extreme diet, major organ dysfunction, cancer, other rheumatic or autoimmune diseases including osteoarthritis, systemic lupus erythematosus, Sjögren syndrome, diabetes were excluded. A total of 79 participants who met the inclusion and exclusion criteria were enrolled, including 29 high-risk individuals, 27 RA patients and 23 HCs. The study procedure was approved by the Biomedical Research Ethics Committee, West China Hospital of Sichuan University (ChiCTR1900022605), and the written consents were obtained from all the participants according to the Declaration of Helsinki. Sociodemographic factors and clinical activity are summarized in Table 1.

**Table 1:** Demographic and clinical features among RA patients, high-risk individuals (Pre) and healthy controls.

**Characteristic**	**RA (*n* = 27)**	**High-risk for RA (*n* = 29)**	**Healthy controls (*n* = 23)**
Age, mean (median) years	51.1 (52)	48.1 (50)	49.5 (49)
Female, %	59	41	57
Disease duration, mean (median) months	17.9 (12.5)	–	–
Disease activity parameter			
ESR, mean (median) mm/h	36.05 (27.50)	–	–
CRP, mean (median) mg/L	14.99(3.18)	–	–
DAS28, mean (median)	4.98 (4.61)	–	–
Autoantibody status			
ACPA positive, *n* (%)	25 (93)	29 (100)	0
IgM-RF positive, *n* (%)	23 (85)	4 (14)	0
ACPA titer, mean (median) U/ml	320.3 (384.3)	173.4 (89.6)	–
IgM-RF titer, mean (median) IU/ml	281.4 (126.0)	47.1 (20.0)	–
Medication use, %			
DMARDs (MTX, LEF, HCQ)	81	0	0
Prednisone	74	0	0
Biologic agent	0	0	0
Smoking status, *n* (%)			
Current	8 (30)	12 (42)	8 (35)
Former	3 (12)	2 (7)	1 (4)
Never	16 (58)	15 (51)	14 (61)
Periodontitis, %^*^	63	62	57

*ESR, erythrocyte sedimentation rate; CRP, C-reactive protein; DAS28, Disease Activity Score in 28 joints; ACPA, anti–citrullinated protein antibody; IgM-RF, IgM rheumatoid factor; DMARDs, Disease-modifying antirheumatic drugs; MTX, Methotrexate; LEF, Leflunomide; HCQ, hydroxychloroquine*.

* *Periodontitis was assessed using a self-reported questionnaire involving bleeding on brushing teeth, non-traumatic loose or missing teeth, or periodontal disease diagnosed by a dentist. Individuals reporting any of these issues were recorded positive*.

### Periodontal Health Evaluation and Saliva Collection

Periodontitis was assessed using a self-reported questionnaire involving bleeding on brushing teeth, non-traumatic loose or missing teeth, or periodontal disease diagnosed by a dentist. Individuals reporting any of these issues were recorded positive. Participants were asked to refrain from eating, drinking or smoking for 30 min prior to sample collection. For saliva collection, participants were first asked to rinse mouth with bottled water to remove food debris, keep lips shut for 3 min, and then spit saliva directly into a 50ml sterile Falcon tube (Becton). After collection, samples were immediately frozen and stored at −80°C.

### Bacterial DNA Extraction

Microbial DNA was isolated from saliva using the FastDNA^®^ SPIN Kit for Soil and the FastPrep^®^ Instrument (MP Biomedicals, Santa Ana, CA) according to manufacturer's protocols. Concentration and purification of the final DNA were determined by NanoDrop 2000 (Thermo Scientific, USA), and quality checked by 1% agarose gel electrophoresis. The V3-V4 hypervariable regions of the bacteria 16S rRNA gene were amplified with primers 338F (5′-ACTCCTACGGGAGGCAGCAG-3′) and 806R (5′-GGACTACHVGGGTWTCTAAT-3′) by thermocycler PCR system (GeneAmp 9700, ABI, USA). The resulted PCR products were extracted from a 2% agarose gel and further purified using the AxyPrep DNA Gel Extraction Kit (Axygen Biosciences, USA) and quantified using QuantiFluor™-ST (Promega, USA) according to the manufacturer's protocol.

### Illumina MiSeq Sequencing and Processing of Sequencing Data

Purified amplicons were pooled in equimolar and paired-end sequenced on an Illumina MiSeq platform (Illumina, San Diego, USA). Raw fastq files were quality-filtered by Trimmomatic and merged by FLASH with the following criteria: (i) the reads were truncated at any site receiving an average quality score <20 over a 50 bp sliding window, (ii) sequences whose overlap being longer than 10 bp were merged according to their overlap with mismatch no more than 2 bp, and (iii) sequences of each sample were separated according to barcodes (exactly matching) and Primers (allowing 2 nucleotide mismatching), and reads containing ambiguous bases were removed. Operational taxonomic units (OTUs) were clustered with 97% similarity cutoff using UPARSE (version 7.1) with a novel “greedy” algorithm that performs chimera filtering and OTU clustering simultaneously. The taxonomy of each 16S rRNA gene sequence was analyzed by RDP Classifier algorithm (http://rdp.cme.msu.edu/) against the Silva (SSU132) 16S rRNA database using confidence threshold of 70%. The fastq files were deposited into the NCBI Sequence Read Archive (SRA) database (Accession Number: PRJNA578951).

### Statistical Analysis

To determine statistically different bacterial taxa among the three groups, we applied the Kruskal-Wallis H-test, with multiple test corrected by Benjamini-Hochberg false discovery rate (FDR) test. *Post-hoc* test was applied to further determine the difference between each group-pair if multiple test among the three groups was significantly different. The linear discriminant analysis (LDA) effect size (LefSe) analysis was applied to detect the most discriminatory taxa among groups. Different features with an LDA score cut-off of 3.0 were identified. For cross-sectional analyses of baseline characteristics and comparison of diversity indexes among three groups, differences were evaluated using the one-way ANOVA test, corrected by FDR. The ANOSIM test was applied to the binary euclidean distance matrix containing all analyzed samples to define if the overall structure of the microbiota was significantly different between the groups. Spearman's correlation analyses were used to assess potentially clinically relevant associations on all taxa. The correlation network between the genera was plotted using cytoscape. Significant correlations with absolute value of Spearman correlation coefficient (rho) >0.5 were plotted. Two-tailed *P* < 0.05 were considered significant.

## Results

### Characteristic of Participants

Detailed demographic characteristics of the participants enrolled in the study are given in Table 1. Age and gender were comparable among the three groups. The mean disease duration in RA patients was 17.9 months (median 12.5 months) and the mean DAS28 was 4.98, reflecting the presence of moderate disease. Nine out of 29 individuals reported having a history of arthralgia in high-risk group, although no arthritis based on physical examination of 44 joints was detected at the time of enrollment. Among RA patients, ACPA and IgM-RF positivity were 93 and 85%, respectively, compared to 100 and 14% in high-risk individuals (ACPA, *P* = 0.23; IgM-RF, *P* < 0.0001). Compared to the high-risk individuals, the sera antibodies titers in RA patients were 1.8-fold higher for ACPA (median level 320.3 vs. 173.4 U/ml) and 6-fold higher for IgM-RF (median level 281.4 vs. 47.1 IU/ml), respectively. Most of the patients with RA were on DMARDs (81%) and/or prednisone (74%) treatment. This study enrolled patients who were naïve to biological agent treatment. Smoking status and periodontitis were not significantly different between groups.

### Distinct Features of Oral Microbial Community in Individuals at High-Risk for RA

**Figure 1:**
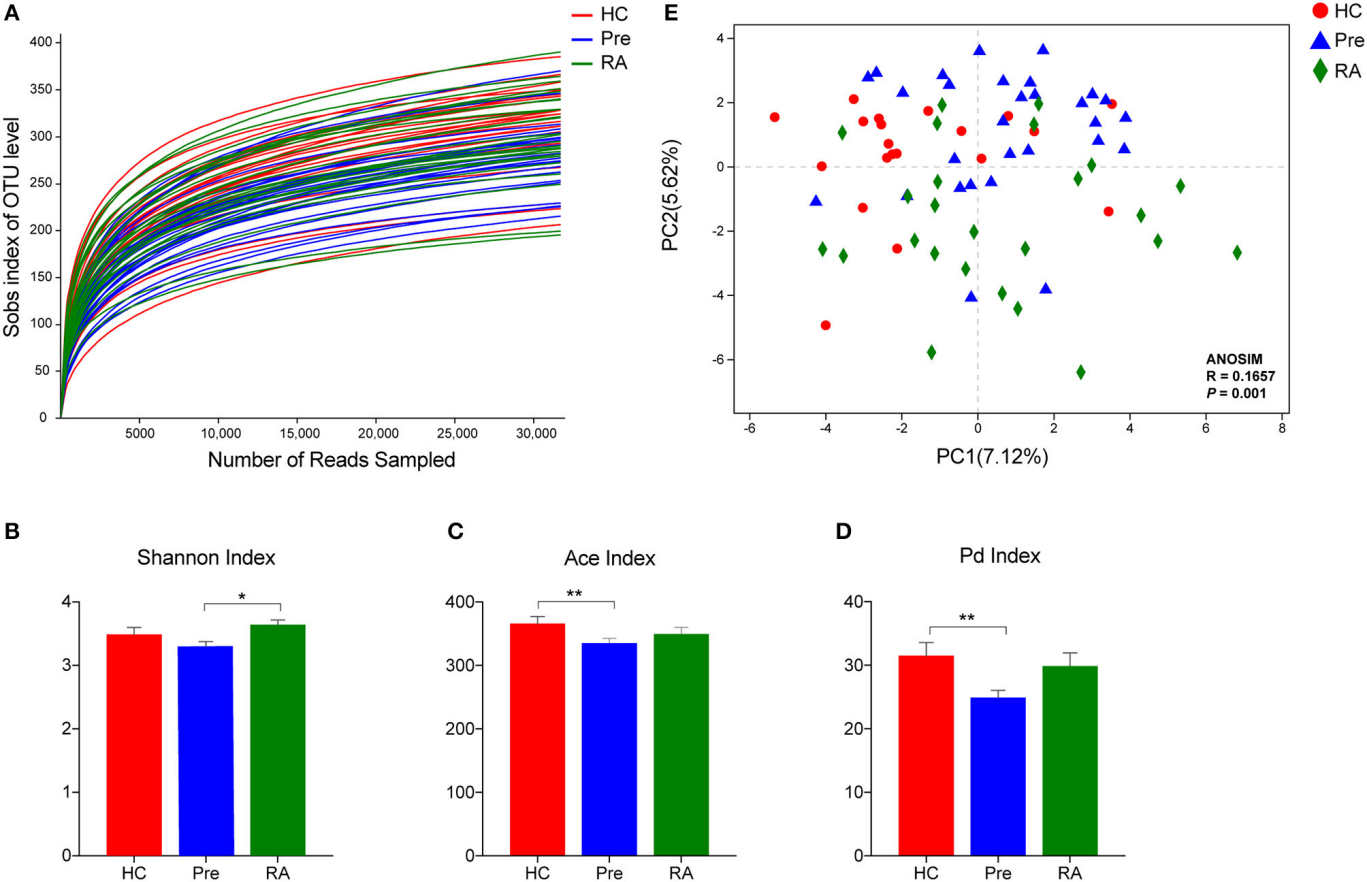
Oral microbiota community characteristics in high-risk individuals (Pre), RA patients and healthy controls (HCs). **(A)** Rarefaction curves showing the number of bacterial sequences obtained are saturated. Alpha diversity was calculated using Shannon diversity index **(B)**, Ace community richness index **(C)**, and the Faith's phylodiversity index **(D)**, revealing a distinct feature in high-risk individuals. **(E)** Beta diversity demonstrated that samples clustered showed a tendency of gradual change from healthy subjects, high-risk individuals to RA patients. *p < 0.05 and **p < 0.01.

Overall, 79 saliva samples yielded 16S rRNA V3-V4 gene sequences with a median depth of sequencing of 53,183 reads per sample (IQR = 45,928–60,223). Using a distance-based similarity of ≥97% for species level OTU assignment, a total of 1,088 OTUs were identified. The number of OTUs from each sample increased sharply before reaching a plateau, which indicates that the number of bacterial sequences obtained represented the bacterial communities well, as the rarefaction curves tended toward saturation (Figure 1A). When compared to HCs and RA patients, oral microbial alpha-diversity was significantly reduced in high-risk individuals (designated “Pre”), as shown by the Shannon diversity index (Pre vs. RA), the ace community richness index and Faith's phylodiversity index (Pre vs. HCs) (Figures 1B–D). Subsequently, we analyzed whether the overall structure of the oral microbiota of HCs differed from that of RA and at-risk individuals based on binary euclidean distance. We further applied PCoA analysis to cluster samples along orthogonal axes of maximal variance. As shown in Figure 1E, beta-diversity plots differentiated the oral microbiota of HCs, RA patients and high-risk individuals (ANOSIM test; *R* = 0.1657, *P* = 0.001).

### Alteration of Specific Taxa Abundance in High-Risk Individuals

**Figure 2:**
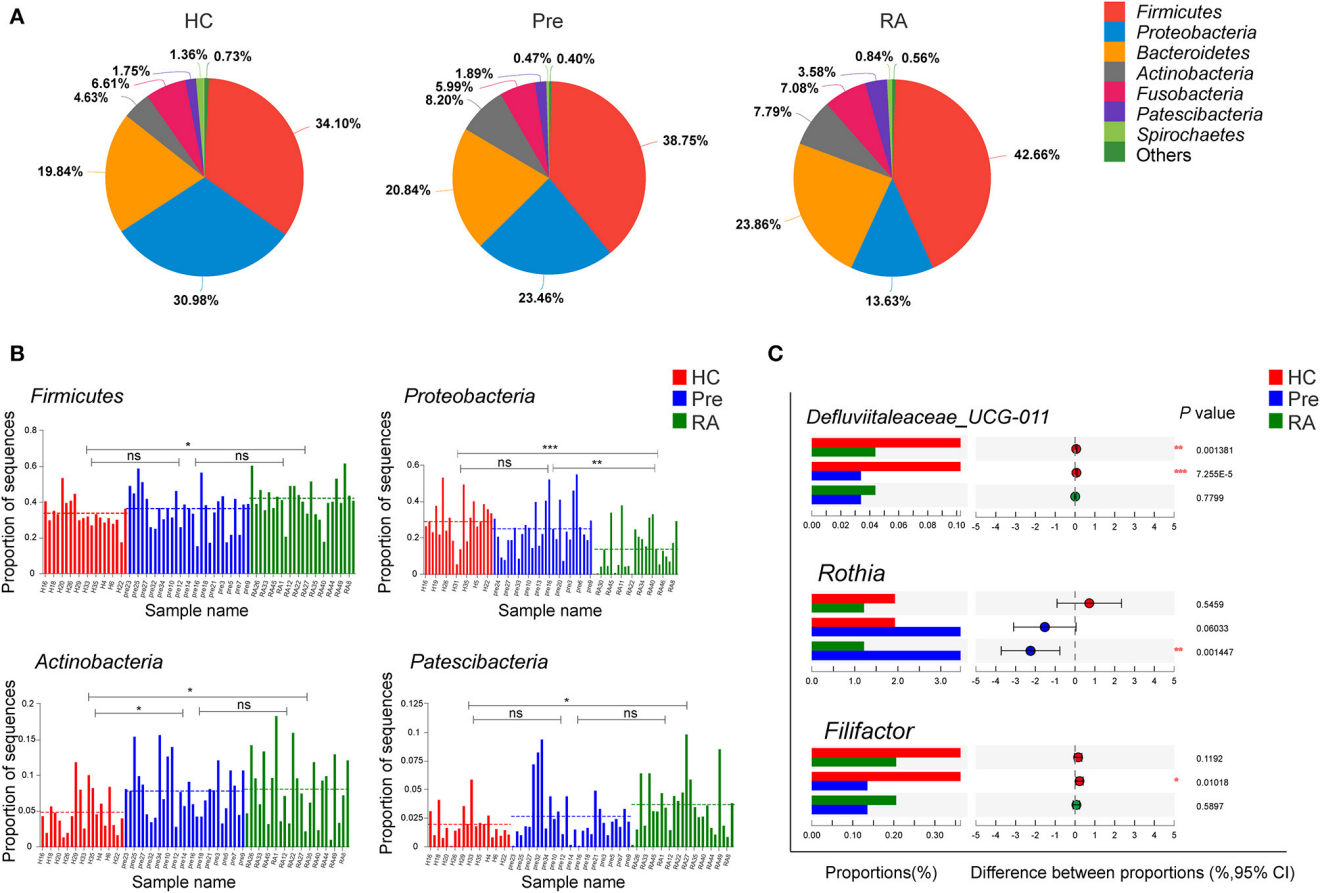
Differentially abundant taxa at phylum and genus level. **(A,B)** The relative abundance of dominant phyla dynamically altered at different stages of RA as represented by healthy controls (HCs), high-risk for RA individuals (Pre), and RA patients (RA). **(C)** Characteristic genera changes were found in high-risk individuals. *p < 0.05; **p < 0.01; and ***p < 0.001; ns, non-significant.

To further probe the distinct bacterial taxa among groups, we first analyzed the relative abundance of the most abundant taxa. The pie charts revealed that the dominant phyla across all subjects were *Firmicutes, Proteobacteria, Bacteroidetes, Fusobacteria*, and *Actinobacteria*, which together accounted for more than 95% of bacterial sequences (Figure 2A). We then analyzed the differential phyla among the three groups. Notably, the relative abundance of *Firmicutes* increased gradually from HCs, high-risk individuals to RA patients (Figures 2A,B, *P* = 0.0421 for RA vs. HCs), while *Proteobacteria* showed an inverse trend of transition (Figures 2A,B, *P* = 0.0003199 for RA vs. HCs). The ratio of *Firmicutes* to *Proteobacteria* significantly increased from HCs to high-risk individuals to RA patients as evaluated by Chi-square test (Supplementary Figure 1, *P* = 0.0083). Other taxa including *Actinobacteria* and *Patescibacteria* showed a similar trend of enrichment in high-risk individuals and RA patients vs. HCs (Figures 2A,B).

**Figure 3:**
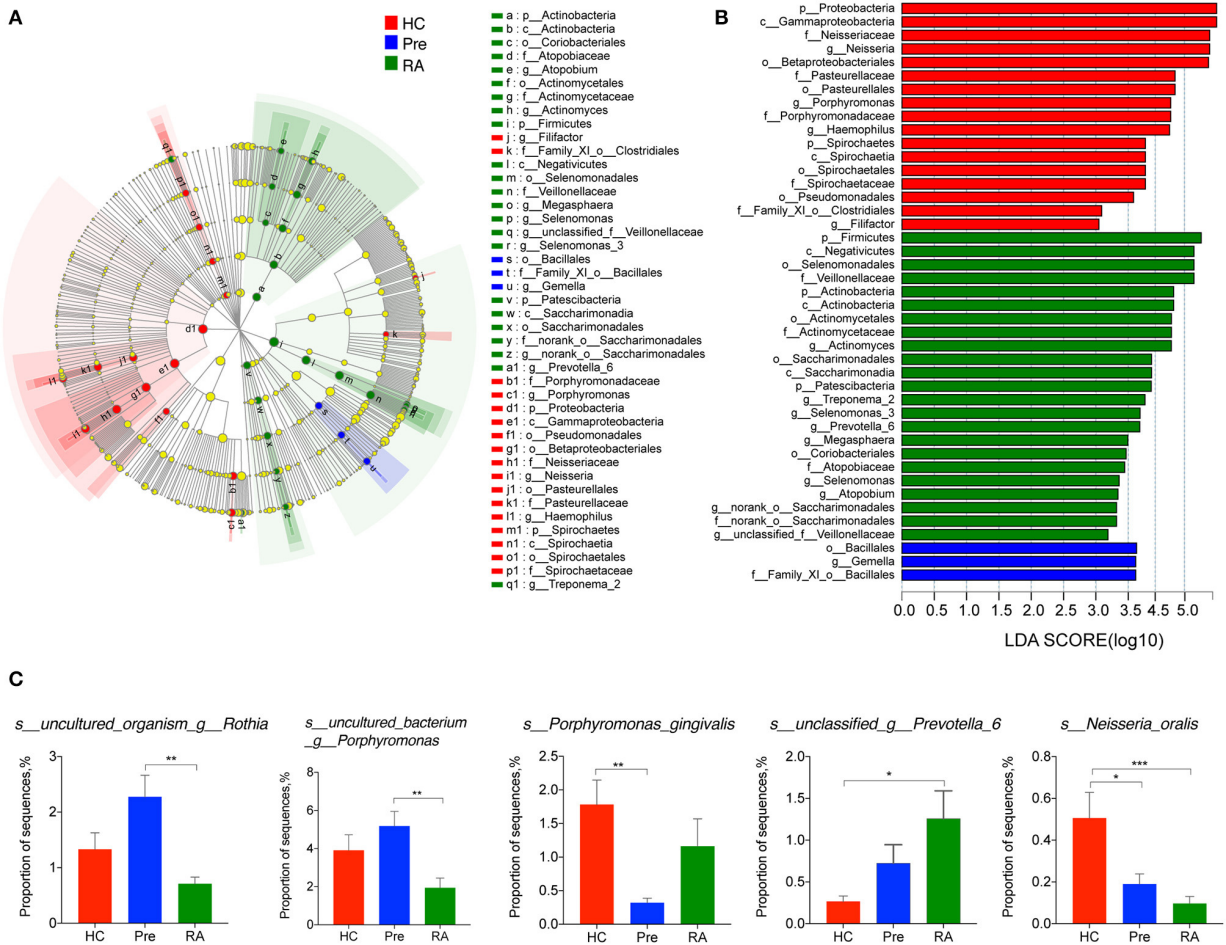
LEfSe analysis revealed the specific taxa changes in high-risk individuals (Pre) and RA patients. LefSe analysis was applied to identify differentially abundant taxa, which are highlighted on the phylogenetic tree in cladogram format **(A)** and for which the LDA scores more than 3 are shown **(B)**. **(C)** Species abundance changes unique to at-risk individuals, and those consistent with changes in RA patients. LDA, linear discriminant analysis; LefSe, the LDA effect size. *p < 0.05; **p < 0.01; and ***p < 0.001.

We then applied Kruskal-Wallis followed by FDR correction and the LEfSe method to analyze more specific differences in microbiota composition among three groups. At the genus level, *Defluviitaleaceae_UCG-011* was significantly decreased in both RA and high-risk individuals compared to HCs (Figure 2C). Characteristic genera changes were found in high-risk individuals, with *Rothia* genus increased (Pre vs. RA) and *Filifactor* decreased (Pre vs. HCs, Figure 2C). The abundance of three genera, *Actinomyces, Prevotella_6*, and *Parvimonas* showed tendencies of gradual change in different stages of disease represented by the three groups, although significant differences were achieved only between RA patients and HCs. The genera enriched in RA patients compared to HCs included *Actinomyces, Prevotella_6*, and *Selenomonas_3*, whilst *Neisseria, Haemophilus, Parvimonas*, and *Eubacterium_yurii_group* were diminished in RA patients (Supplementary Figure 2). These findings were further verified by the LEfSe analysis, as shown in the cladogram and the contributory discriminate taxa with LDA score >3 was plotted for each group (Figures 3A,B).

The titres of antibodies to a periodontopathic species, *P. gingivalis*, are associated with the presence of RA-related autoantibodies in at-risk individuals (Mikuls et al., 2012; Johansson et al., 2016). A further study indicates *P. gingivalis* not only induced periodontitis, but also worsened the concurrent T cell-dependent arthritis in mice (de Aquino et al., 2014). We, therefore, assumed an elevated abundance of *P. gingivalis* in the saliva of high-risk individuals and RA patients. Contrary to our initial hypothesis, there was no significant difference in its relative abundance in RA patients compared to HCs. Moreover, the relative abundance of *P. gingivalis* was significantly decreased in high-risk individuals (Pre vs. HCs, Figure 3C). We also found two uncultured species within the *Rothia* genus and *Porphyromonas* genus more abundant in high-risk individuals than in RA patients. Intriguingly, among other differentially abundant species in RA patients (Supplementary Figure 3), an unclassified species annotated within the *Prevotella_6* genus was elevated in the saliva of RA patients (RA vs. HCs, *P* = 0.009). The relative abundance of *Neisseria oralis* was decreased in both RA patients and high-risk individuals (Figure 3C).

### Systemic Autoimmune Signature in High-Risk Individuals and RA Patients Is Associated With Characteristic Saliva Taxa

To further investigate whether the observed changes in saliva microbiota was associated with the autoimmune characteristics in high-risk individuals and with other disease parameters in RA, we analyzed the correlations of the relative abundance of bacterial genera with (1) serum concentrations of ACPA and RF in high-risk and RA individuals, (2) disease activity parameters including CRP, ESR, DAS28, and (3) course of the disease in RA patients.

**Figure 4:**
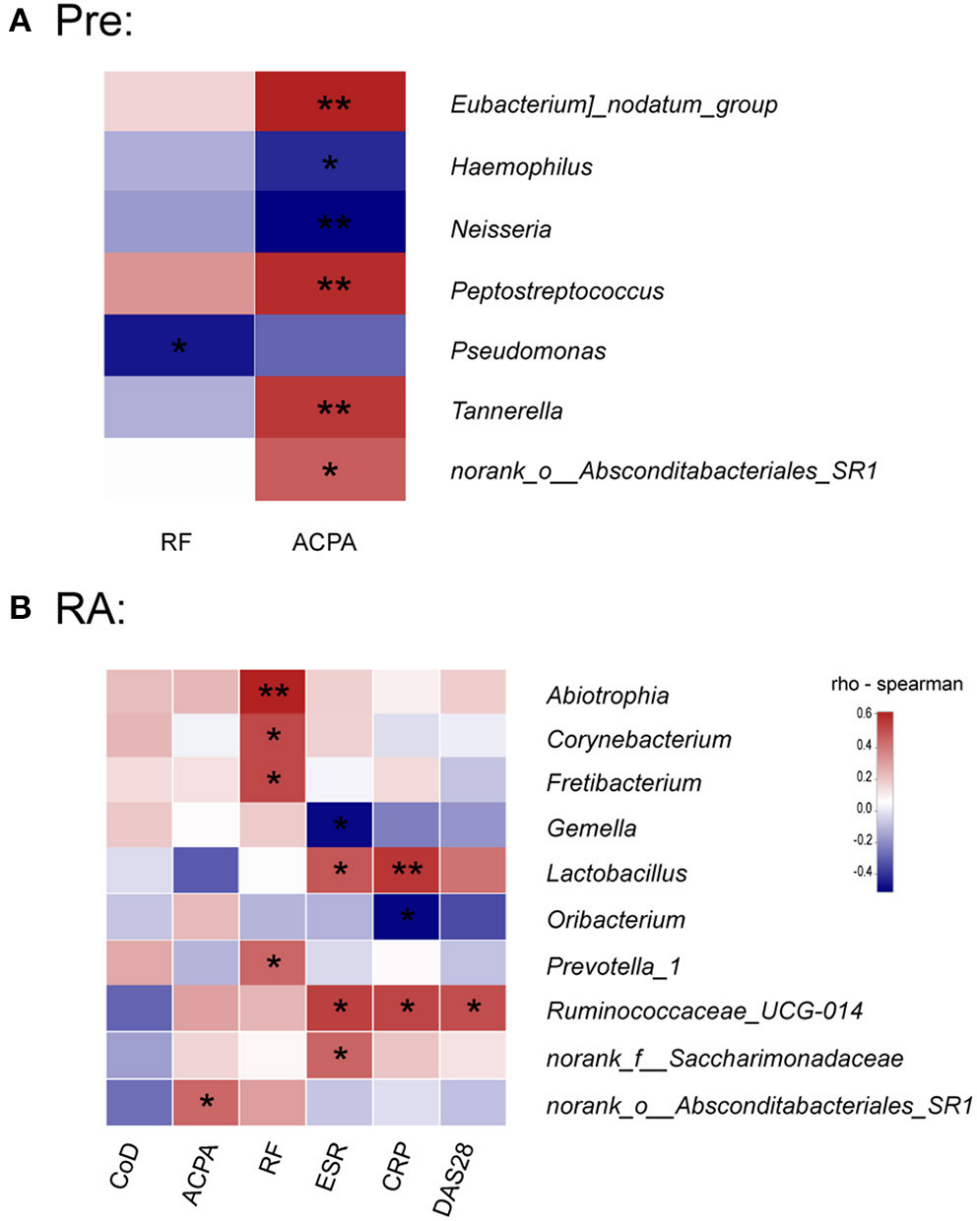
Association between saliva microbiota abundance and systemic autoimmune signature in high-risk individuals and RA patients. **(A,B)** The correlations between the relative abundance of saliva bacterial genera with serum concentrations of ACPA and RF in high-risk individuals and RA patients. **(B)** Correlations between the relative abundance of specific genera and serum ACPA and RF concentrations, disease activity score (DAS28), serum acute phase reactants levels, and course of the disease (CoD) in RA patients. The color scale represents the magnitude of correlation. Red scale indicates positive correlations; blue scale indicates negative correlations. Pre, “pre-clinical” at risk for RA individuals; RF, rheumatoid factor; ACPA, anti-citrullinated protein antibodies; CRP, C-reactive protein; ESR, erythrocyte sedimentation rate. *p < 0.05 and **p < 0.01.

The study revealed that in high-risk individuals, serum ACPA concentration was positively correlated with the relative abundance of *Eubacterium nodatum_group, Peptostreptococcus, Tannerella, norank_o__Absconditabacteriales_SR1*, while conversely associated with *Haemophilus* and *Neisseria* (Figure 4A), both of which were significantly decreased in RA patients (Supplementary Figure 2). A negative correlation with *Pseudomonas* was found for RF in high-risk individuals (Figure 4A).

In RA patients, however, no genus was correlated with the disease course, which indicates a relatively stable saliva bacterial community in the time spans (Figure 4B). Contrary to the study in high-risk individuals, only *norank_o__Absconditabacteriales_SR1* was positively associated with serum ACPA concentration in RA patients. Notably, the concentration of RF was significantly associated with the relative abundance of *Abiotrophia, Corynebacterium, Fretibacterium*, and *Prevotella_1*. Clinical disease activity parameter DAS28 positively correlated with *Ruminococcaceae_UCG-014*, which was also associated with CRP and ESR. In addition, *Lactobacillus* and *norank_f__Saccharimonadaceae*, both increased in RA patients (Supplementary Figure 2), positively correlated with ESR (Figure 4B). Finally, CRP value at the time of enrollment was also positively associated with *Lactobacillus* and negatively with *Oribacterium*.

### Examination of Interactions Among Differentially Abundant Microbes

**Figure 5:**
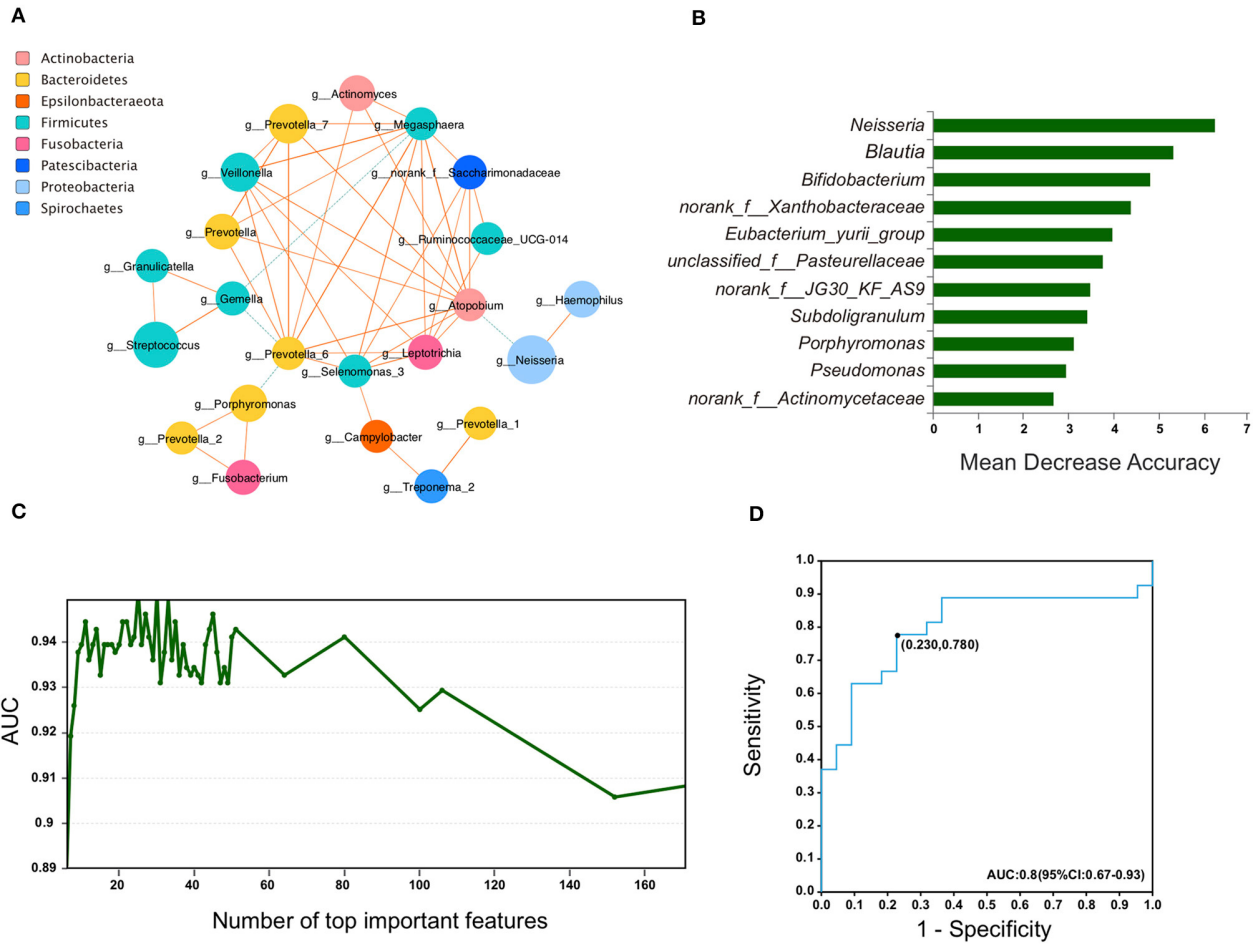
Saliva bacterial biomarkers characterizes RA patients. **(A)** Plots of co-abundance and co-exclusion association networks between differentially abundant genera. Each node represents one genus. The node size is proportional to the mean relative abundance of the genus in all samples. Node color indicates the phylum it belongs to. Lines between nodes show positive correlations (solid orange lines) or negative correlations (dashed green lines). Line width is proportional to the value of Spearman correlation coefficient and reflects the magnitude of association. **(B–D)** A random forest model was applied to identify bacterial biomarkers for RA patients. Ranked lists of genera in order of random forests reporting feature importance scores were obtained **(B)** and an AUC-validation method was used to determine the optimal genera set **(C)**. The built ROC curve based on the selected panel of 11 genera yield an AUC of 0.8 **(D)**.

A correlation network was constructed to investigate the co-abundance and co-exclusion interactions between the differentially abundant microbes. The correlated genera were from eight phyla as indicated in Figure 5A. Most of the correlations within the community were positive, with only a few negative correlations. *Gemella* genus was negatively associated with *Megasphaera* and *Prevotella_6*. *Prevotella_6*, on the other side, was also negatively associated with *Porphyromonas*. Additionally, significant negative correlation was found between *Atopobium* and *Neisseria*.

### Models of Saliva Bacterial Biomarkers Profile and Predicted Function in RA Patients

We next used the machine learning random forests algorithm to construct a prediction model (Breiman, 2001). A panel of 11 genera was selected based on the model (Figures 5B,C). The efficacy of these differentially expressed bacteria in discriminating between RA patients and HCs was calculated using a receiver operator characteristic (ROC) curve. The area under the ROC curve (AUC) was 80.0%, and the 95% confidence interval (CI) was 67–93% (Figure 5D).

We then applied PICRUSt (Langille et al., 2013) to infer the functional content of the microbiota. The associations between differentially abundant taxa with predicted functional pathways KEGG (Kyoto Encyclopedia of Genes and Genomes) was illustrated in Supplementary Figure 4A. Specifically, an increase in OTUs functional representatives of the bacterial toxins pathway, carbohydrate digestion and absorption pathway, starch, and sucrose metabolism pathway was observed in RA patients compared to HCs. By comparison, OTUs involved in fatty acid biosynthesis, glutathione metabolism, glycan biosynthesis and metabolism, inorganic ion transport and metabolism, and phosphatidylinositol signaling system were decreased in RA patients (Supplementary Figure 4B).

## Discussion

Our study identified, for the first time, compositional alterations of oral microbiota in individuals at high-risk for RA, who have developed systemic autoimmunity associated with RA. Importantly, several saliva taxa changes were associated with serum autoantibody levels or clinical disease parameters in high-risk individuals and/or RA patients. A bacterial biomarker panel was constructed in discriminating RA patients from healthy individuals. Putative markers involved in bacterial toxins pathway, carbohydrate digestion and absorption were upregulated while pathways involved in fatty acid biosynthesis, glycan biosynthesis and metabolism, and phosphatidylinositol signaling were under-represented in RA patients. These findings support the hypothesis that microbiome changes occurring in mucosal sites such as the oral cavity might contribute to disease pathogenesis in the initial stages of RA.

We found that the microbial diversity in the oral cavity was comparable between HCs and RA patients. This is in line with a prior study that reported similar microbial richness and diversity in subgingival samples from new-onset RA patients (NORA) and HCs (Scher et al., 2012). Similar results were also reported in stool samples from RA patients and HCs (Zhang et al., 2015). Noticeably, we identified that in “pre-clinical” high-risk individuals, microbial diversity and richness was significantly reduced compared to RA patients and HCs. The overall composition of saliva microbial communities was also different amongst the three groups, suggesting oral microbiota perturbations already exist in the “pre-clinical” stage of RA.

Although some genera were significantly altered in RA patients (discussed later), only a few genera characterized the oral microbiome of high-risk individuals, including *Rothia* and *Filifactor*. Of note, some species of the genus *Rothia* have been identified as opportunistic pathogens and can cause bacteremia, endocarditis, joint infections, and pneumonia (Boudewijns et al., 2003; Verrall et al., 2010; Ramanan et al., 2014; de Steenhuijsen Piters et al., 2016). We found that the *Rothia* genus, and an uncultured species annotated within the genera were enriched in high-risk individuals compared to RA patients. Interestingly, the *Filifactor* genus (including *Filifactor alocis* and an unclassified species) was diminished in high-risk individuals. In fact, *F. alocis* has been associated with periodontitis and periodontal biofilm formation. Similarly, even though suspicions exist that the PAD-producing *P. gingivalis* might be involved in RA pathogenesis (Mikuls et al., 2012; de Aquino et al., 2014), our study found comparable level of *P. gingivalis* in RA patients and HCs and no association of this species with autoantibody titer was observed. This finding is in agreement with recent studies that did not find an association between *P. gingivalis* or its PAD and RA (Scher et al., 2012; Konig et al., 2015). Interestingly, we found decreased level of *P. gingivalis* in high-risk individuals, even though periodontitis prevalence was similar to that in HCs. The unique oral microbial features in high-risk individuals reflect the characteristics of the pre-clinical stage.

As expected, only a minority of taxa changes characterized the high-risk stage while most of the significant discrepancies were only found between the diagnosed RA patients and HCs. Importantly, some taxa exhibited a tendency of gradual change during the development stages of the disease, including three genera *Actinomyces, Prevotella_6, Parvimonas* and an unclassified species within *Prevotella_6*. Indeed, the enrichment of *Actinomyces* and *Prevotella* have been reported in RA patients' oral samples (Scher et al., 2012; Zhang et al., 2015). Recently, much attention has been paid to the potential pathogenic role of *Prevotella* spp., especially an intestinal species *Prevotella* copri, in RA. Presence of *P. copri* in fecal samples was strongly correlated with disease in NORA (Scher et al., 2013). A recent study further highlighted the association of the *Prevotella* spp. with RA pathogenesis by revealing its enrichment in fecal sample of “pre-clinical” RA individuals (Alpizar-Rodriguez et al., 2019). Our study identified the increase of *Prevotella_6* in saliva samples with disease progression, which further indicates that *Prevotella*, not limited to the gut but also other mucosal sites, may be involved in RA pathogenesis. Additionally, consistent with a previous study in DMARDs naïve RA patients (Zhang et al., 2015), both *Haemophilus* spp. and *Neisseria* spp. were depleted in our group of RA patients. Intriguingly, although not significantly reduced compared to HCs, the relative abundance of both genera negatively correlated with the level of serum ACPA in high-risk individuals. Further, in line with the study by Zhang et al. (2015), *Lactobacillus* spp. was elevated in saliva of our RA patients, and its level was positively correlated with acute inflammation makers such as CRP and ESR. Given these tantalizing clues indicating a contribution of microbial dysbiosis at various mucus sites to RA pathogenesis, it is likely that complex microbiome network in multiple mucus sites align together in this process.

However, our study has limitations. The oral cavity is home to one of the most diverse microbial communities of human body, with the flowing saliva containing the “whole-mouth” bacterial community and most commonly studied (Mascitti et al., 2019). However, bacteria colonize all sites within the oral cavity, forming different habitats with non-overlapping microbial populations. Regional variations, such as the bacterial profiles in the supragingival plaque of tooth surfaces and subgingival plaque, etc., were not investigated. Samples from multiple oral sites may help build a panoramic view of the oral microbiome association with the disease (Mascitti et al., 2019). Additionally, relatively small number of participants limits the statistical power of the study, given that samples from the individuals in pre-clinical stages is difficult to acquire. A larger study including more participants would help draw a more concrete conclusion. Another limitation is that most of the involved established RA patients were under treatment, which may affect the salivary microbiome of RA patients. Even though we have a main study focus on the high-risk individuals, who were treatment-naïve, an also treatment-naïve group of RA patients would help improve the study. Finally, the assessment of periodontitis was based on self-reported questionnaires regarding periodontitis symptoms and medical history. Periodontitis status might be underestimated or overestimated in an individual. However, as the questionnaire was applied to all individuals independently, a presumably equal readout would be expected. Further, our study didn't find an association between the abundance of specific periodontitis pathogen with autoantibody titer in either high-risk individuals or RA patients. The seemingly paradoxical finding of lower abundance of periodontitis associated pathogen *P. gingivalis* in high-risk individuals complicates what is known about the relationship between periodontitis and RA pathogenesis. Further studies involving larger number of pre-clinical individuals and graded severity of periodontitis assessment would help better clarify this association.

## Conclusions

In summary, we demonstrate that the pre-clinical stage of RA is characterized by oral dysbiosis, some of which are correlated with autoantibody titer. Common and unique microbial features exist in the high-risk stage compared to established RA. Our findings support the mucosal origin hypothesis in the development of RA. Further mechanistic insights into possible causation through well-designed prospective human studies and evidence derived from *in vivo* experiments from animal models are warranted.

## Data Availability Statement

The fastq files were deposited into the NCBI Sequence Read Archive (SRA) database (Accession Number: PRJNA578951).

## Ethics Statement

The studies involving human participants were reviewed and approved by Biomedical Research Ethics Committee, West China Hospital of Sichuan University. The patients/participants provided their written informed consent to participate in this study.

## Author Contributions

YLuo and YLiu designed and supervised the study, reviewed and edited the manuscript. YT, LZ, PQ, HZ, QZ, and YZ collected the samples and sociodemographic, and pathological data. YT, YLi, and LS curated the data. YT, LZ, and YLuo conducted analyses and wrote the first draft of the manuscript. All authors contributed to manuscript revision, read, and approved the submitted version.

### Conflict of Interest

The authors declare that the research was conducted in the absence of any commercial or financial relationships that could be construed as a potential conflict of interest.

## References

[B1] Alpizar-Rodriguez D. Lesker T. R. Gronow A. Gilbert B. Raemy E. Lamacchia C.. (2019). *Prevotella* copri in individuals at risk for rheumatoid arthritis. Ann. Rheum. Dis. 78, 590–593. 10.1136/annrheumdis-2018-214514 30760471

[B2] Berglin E. Padyukov L. Sundin U. Hallmans G. Stenlund H. Van Venrooij W. J.. (2004). A combination of autoantibodies to cyclic citrullinated peptide (CCP) and HLA-DRB1 locus antigens is strongly associated with future onset of rheumatoid arthritis. Arthritis Res. Ther. 6, R303–R308. 10.1186/ar1187 15225365 PMC464874

[B3] Boudewijns M. Magerman K. Verhaegen J. Debrock G. Peetermans W. E. Donkersloot P.. (2003). Rothia dentocariosa, endocarditis and mycotic aneurysms: case report and review of the literature. Clin. Microbiol. Infect. 9, 222–229. 10.1046/j.1469-0691.2003.00503.x 12667255

[B4] Breiman L. (2001). Random forests. Mach. Learn. 45, 5–32. 10.1023/A:1010933404324

[B5] de Aquino S. G. Abdollahi-Roodsaz S. Koenders M. I. van de Loo F. A. Pruijn G. J. Marijnissen R. J.. (2014). Periodontal pathogens directly promote autoimmune experimental arthritis by inducing a TLR2- and IL-1-driven Th17 response. J. Immunol. 192, 4103–4111. 10.4049/jimmunol.1301970 24683190

[B6] de Steenhuijsen Piters W. A. Huijskens E. G. Wyllie A. L. Biesbroek G. van den Bergh M. R. Veenhoven R. H.. (2016). Dysbiosis of upper respiratory tract microbiota in elderly pneumonia patients. ISME J. 10, 97–108. 10.1038/ismej.2015.99 26151645 PMC4681870

[B7] Hirschfeld J. Roberts H. M. Chapple I. L. Parcina M. Jepsen S. Johansson A.. (2016). Effects of *Aggregatibacter actinomycetemcomitans* leukotoxin on neutrophil migration and extracellular trap formation. J. Oral Microbiol. 8:33070. 10.3402/jom.v8.33070 27834173 PMC5103672

[B8] Johansson L. Sherina N. Kharlamova N. Potempa B. Larsson B. Israelsson L.. (2016). Concentration of antibodies against *Porphyromonas gingivalis* is increased before the onset of symptoms of rheumatoid arthritis. Arthritis Res. Ther. 18:201. 10.1186/s13075-016-1100-4 27605245 PMC5015325

[B9] Jorgensen K. T. Wiik A. Pedersen M. Hedegaard C. J. Vestergaard B. F. Gislefoss R. E.. (2008). Cytokines, autoantibodies and viral antibodies in premorbid and postdiagnostic sera from patients with rheumatoid arthritis: case-control study nested in a cohort of Norwegian blood donors. Ann. Rheum. Dis. 67, 860–866. 10.1136/ard.2007.073825 17644543

[B10] Konig M. F. Abusleme L. Reinholdt J. Palmer R. J. Teles R. P. Sampson K.. (2016). Aggregatibacter actinomycetemcomitans-induced hypercitrullination links periodontal infection to autoimmunity in rheumatoid arthritis. Sci. Transl. Med. 8:369ra176. 10.1126/scitranslmed.aaj1921 27974664 PMC5384717

[B11] Konig M. F. Paracha A. S. Moni M. Bingham C. O. 3rd Andrade F. (2015). Defining the role of *Porphyromonas gingivalis* peptidylarginine deiminase (PPAD) in rheumatoid arthritis through the study of PPAD biology. Ann. Rheum. Dis. 74, 2054–2061. 10.1136/annrheumdis-2014-205385 24864075 PMC4368502

[B12] Langille M. G. Zaneveld J. Caporaso J. G. McDonald D. Knights D. Reyes J. A.. (2013). Predictive functional profiling of microbial communities using 16S rRNA marker gene sequences. Nat. Biotechnol. 31, 814–821. 10.1038/nbt.2676 23975157 PMC3819121

[B13] Lappin D. F. Apatzidou D. Quirke A. M. Oliver-Bell J. Butcher J. P. Kinane D. F.. (2013). Influence of periodontal disease, *Porphyromonas gingivalis* and cigarette smoking on systemic anti-citrullinated peptide antibody titres. J. Clin. Periodontol. 40, 907–915. 10.1111/jcpe.12138 23902301

[B14] Mascitti M. Togni L. Troiano G. Caponio V. C. A. Gissi D. B. Montebugnoli L.. (2019). Beyond head and neck cancer: the relationship between oral microbiota and tumour development in distant organs. Front. Cell. Infect. Microbiol. 9:232. 10.3389/fcimb.2019.00232 31297343 PMC6607058

[B15] Mikuls T. R. Thiele G. M. Deane K. D. Payne J. B. O'Dell J. R. Yu F.. (2012). *Porphyromonas gingivalis* and disease-related autoantibodies in individuals at increased risk of rheumatoid arthritis. Arthritis Rheum. 64, 3522–3530. 10.1002/art.34595 22736291 PMC3467347

[B16] Nielen M. M. van Schaardenburg D. Reesink H. W. van de Stadt R. J. van der Horst-Bruinsma I. E. de Koning M. H.. (2004). Specific autoantibodies precede the symptoms of rheumatoid arthritis: a study of serial measurements in blood donors. Arthritis Rheum. 50, 380–386. 10.1002/art.20018 14872479

[B17] Ramanan P. Barreto J. N. Osmon D. R. Tosh P. K. (2014). Rothia bacteremia: a 10-year experience at Mayo Clinic, Rochester, Minnesota. J. Clin. Microbiol. 52, 3184–3189. 10.1128/JCM.01270-14 24951810 PMC4313135

[B18] Rantapaa-Dahlqvist S. de Jong B. A. Berglin E. Hallmans G. Wadell G. Stenlund H.. (2003). Antibodies against cyclic citrullinated peptide and IgA rheumatoid factor predict the development of rheumatoid arthritis. Arthritis Rheum. 48, 2741–2749. 10.1002/art.11223 14558078

[B19] Rombouts Y. Ewing E. van de Stadt L. A. Selman M. H. Trouw L. A. Deelder A. M.. (2015). Anti-citrullinated protein antibodies acquire a pro-inflammatory Fc glycosylation phenotype prior to the onset of rheumatoid arthritis. Ann. Rheum. Dis. 74, 234–241. 10.1136/annrheumdis-2013-203565 24106048

[B20] Ronnelid J. Wick M. C. Lampa J. Lindblad S. Nordmark B. Klareskog L.. (2005). Longitudinal analysis of citrullinated protein/peptide antibodies (anti-CP) during 5 year follow up in early rheumatoid arthritis: anti-CP status predicts worse disease activity and greater radiological progression. Ann. Rheum. Dis. 64, 1744–1749. 10.1136/ard.2004.033571 15843452 PMC1755292

[B21] Schellekens G. A. Visser H. de Jong B. A. van den Hoogen F. H. Hazes J. M. Breedveld F. C.. (2000). The diagnostic properties of rheumatoid arthritis antibodies recognizing a cyclic citrullinated peptide. Arthritis Rheum. 43, 155–163. 10.1002/1529-0131(200001)43:1<155::AID-ANR20>3.0.CO;2-3 10643712

[B22] Scher J. U. Joshua V. Artacho A. Abdollahi-Roodsaz S. Ockinger J. Kullberg S.. (2016). The lung microbiota in early rheumatoid arthritis and autoimmunity. Microbiome 4:60. 10.1186/s40168-016-0206-x 27855721 PMC5114783

[B23] Scher J. U. Sczesnak A. Longman R. S. Segata N. Ubeda C. Bielski C.. (2013). Expansion of intestinal *Prevotella* copri correlates with enhanced susceptibility to arthritis. Elife 2:e01202. 10.7554/eLife.01202 24192039 PMC3816614

[B24] Scher J. U. Ubeda C. Equinda M. Khanin R. Buischi Y. Viale A.. (2012). Periodontal disease and the oral microbiota in new-onset rheumatoid arthritis. Arthritis Rheum. 64, 3083–3094. 10.1002/art.34539 22576262 PMC3428472

[B25] Sokolove J. Bromberg R. Deane K. D. Lahey L. J. Derber L. A. Chandra P. E.. (2012). Autoantibody epitope spreading in the pre-clinical phase predicts progression to rheumatoid arthritis. PLoS ONE 7:e35296. 10.1371/annotation/2e462817-ab93-4d78-95a4-1d8b9d172971 22662108 PMC3360701

[B26] Syversen S. W. Gaarder P. I. Goll G. L. Odegard S. Haavardsholm E. A. Mowinckel P.. (2008). High anti-cyclic citrullinated peptide levels and an algorithm of four variables predict radiographic progression in patients with rheumatoid arthritis: results from a 10-year longitudinal study. Ann. Rheum. Dis. 67, 212–217. 10.1136/ard.2006.068247 17526555

[B27] van de Stadt L. A. van der Horst A. R. de Koning M. H. Bos W. H. Wolbink G. J. van de Stadt R. J.. (2011). The extent of the anti-citrullinated protein antibody repertoire is associated with arthritis development in patients with seropositive arthralgia. Ann. Rheum. Dis. 70, 128–133. 10.1136/ard.2010.132662 21062853

[B28] Verrall A. J. Robinson P. C. Tan C. E. Mackie W. G. Blackmore T. K. (2010). Rothia aeria as a cause of sepsis in a native joint. J. Clin. Microbiol. 48, 2648–2650. 10.1128/JCM.02217-09 20504983 PMC2897501

[B29] Zhang X. Zhang D. Jia H. Feng Q. Wang D. Liang D.. (2015). The oral and gut microbiomes are perturbed in rheumatoid arthritis and partly normalized after treatment. Nat. Med. 21, 895–905. 10.1038/nm.3914 26214836

