# Supplementary material for: Oral Microbiota Perturbations Are Linked to High Risk for Rheumatoid Arthritis
Source: Front Cell Infect Microbiol. 2020 Jan 22;9:475. doi: 10.3389/fcimb.2019.00475 (PMC6987375; doi:10.3389/fcimb.2019.00475)
Supplement: Supplementary file 1 [file Data_Sheet_1.PDF]

Supplementary Material

1. Supplementary Figures

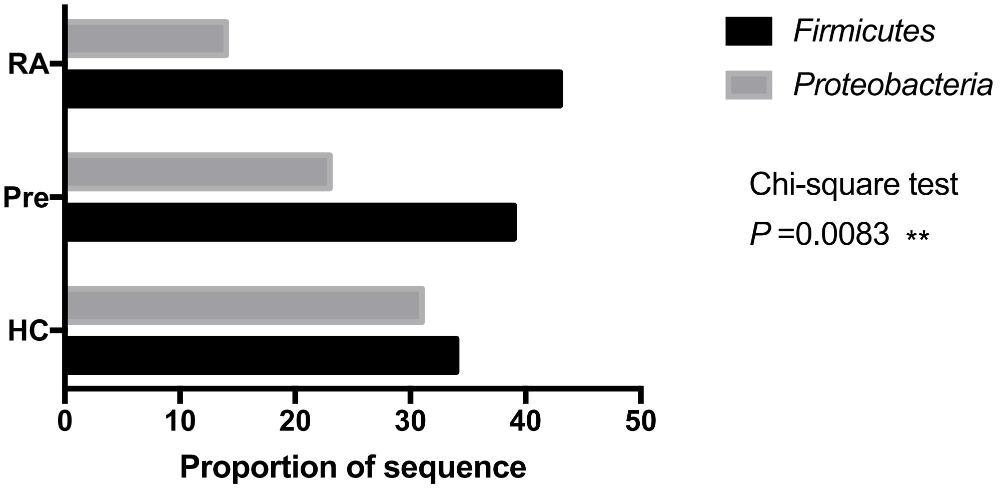

Supplementary Figure 1. Ratio changes of Firmicutes to Proteobacteria (F/P ratio). The relative abundance of *Firmicutes* to *Proteobacteria* (F/P ratio) increases significantly from healthy individuals, high-risk individuals to RA patients. The changes were examined by Chi-square test. RA, established rheumatoid arthritis patients; Pre, high-risk for RA individuals; HC, healthy controls.

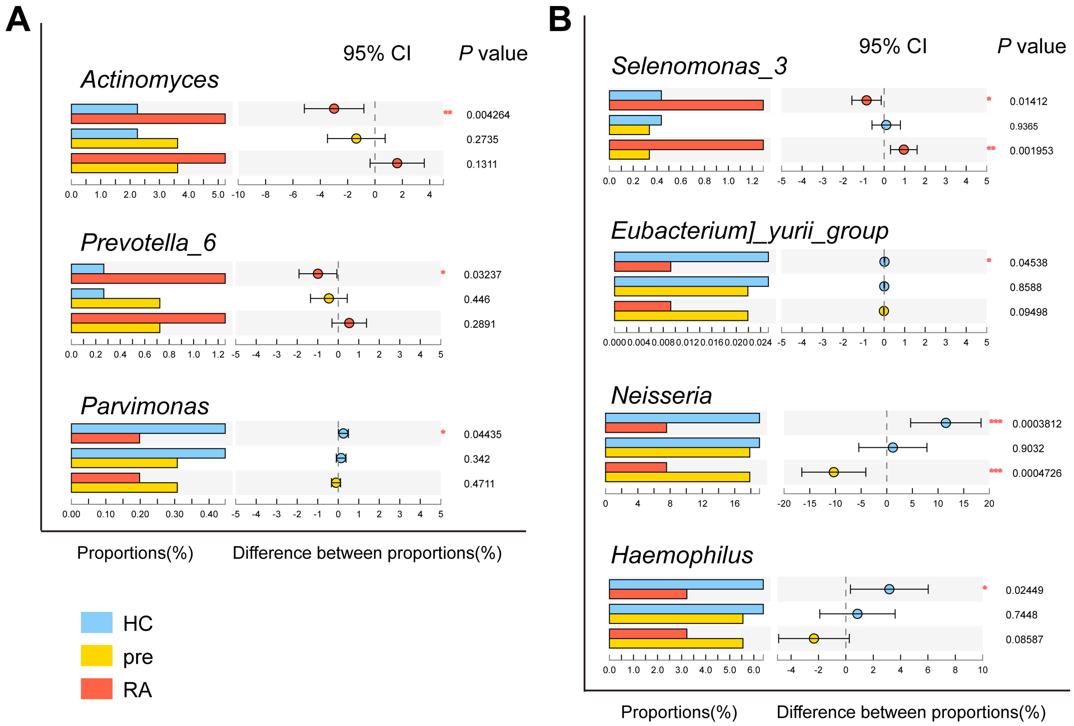

Supplementary Figure 2. Differentially abundant taxa at genus level. Additional genera that have differential abundance among three groups. (A) the abundances of the three genera show tendencies of gradual change in different stages of disease, although significant differences were achieved only between RA patients and healthy individuals. (B) the abundances of these genera are significantly altered only in established RA patients.

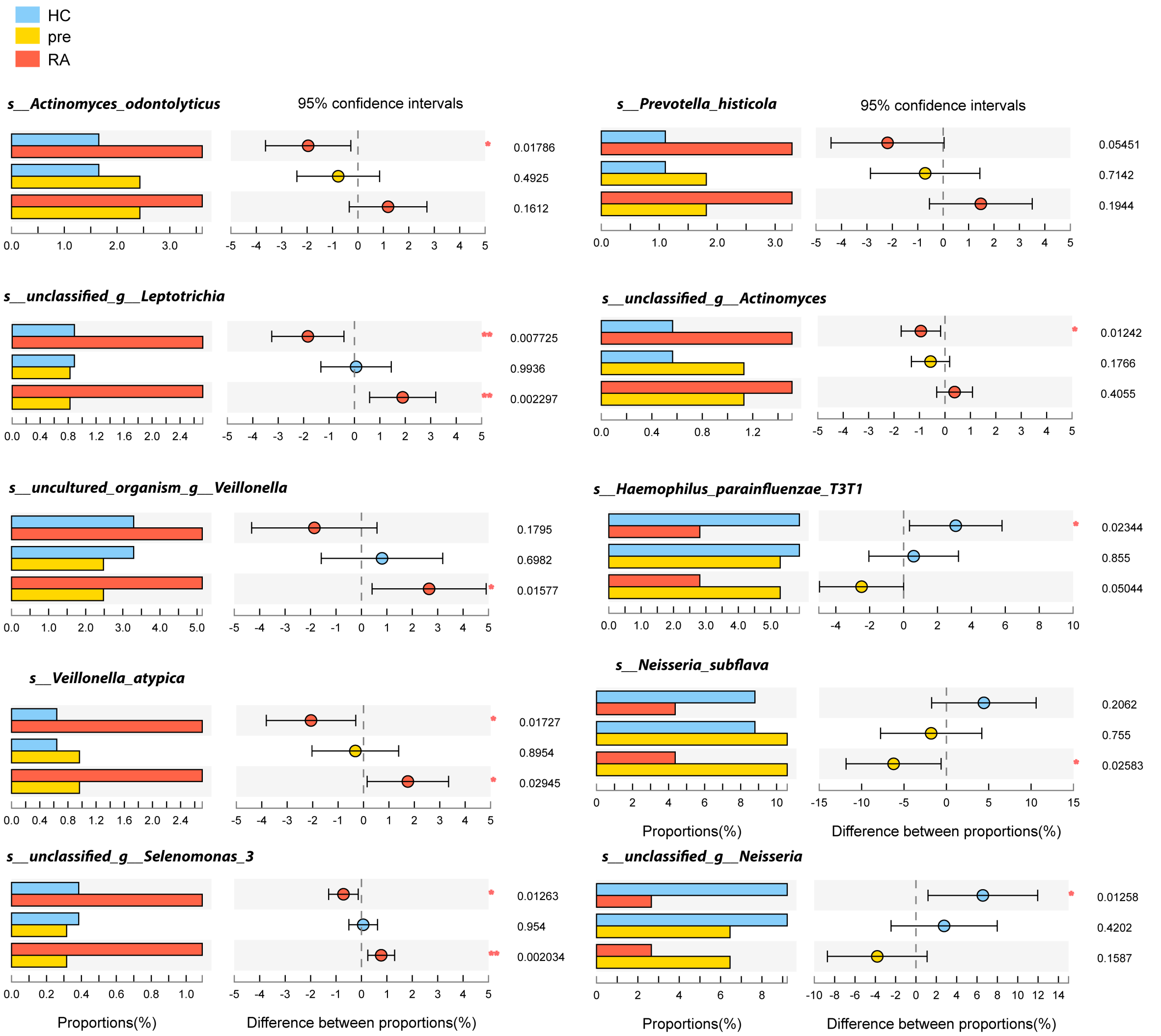

Supplementary Figure 3. Differentially abundant taxa at species level. Additional species that have differential abundance among three groups.

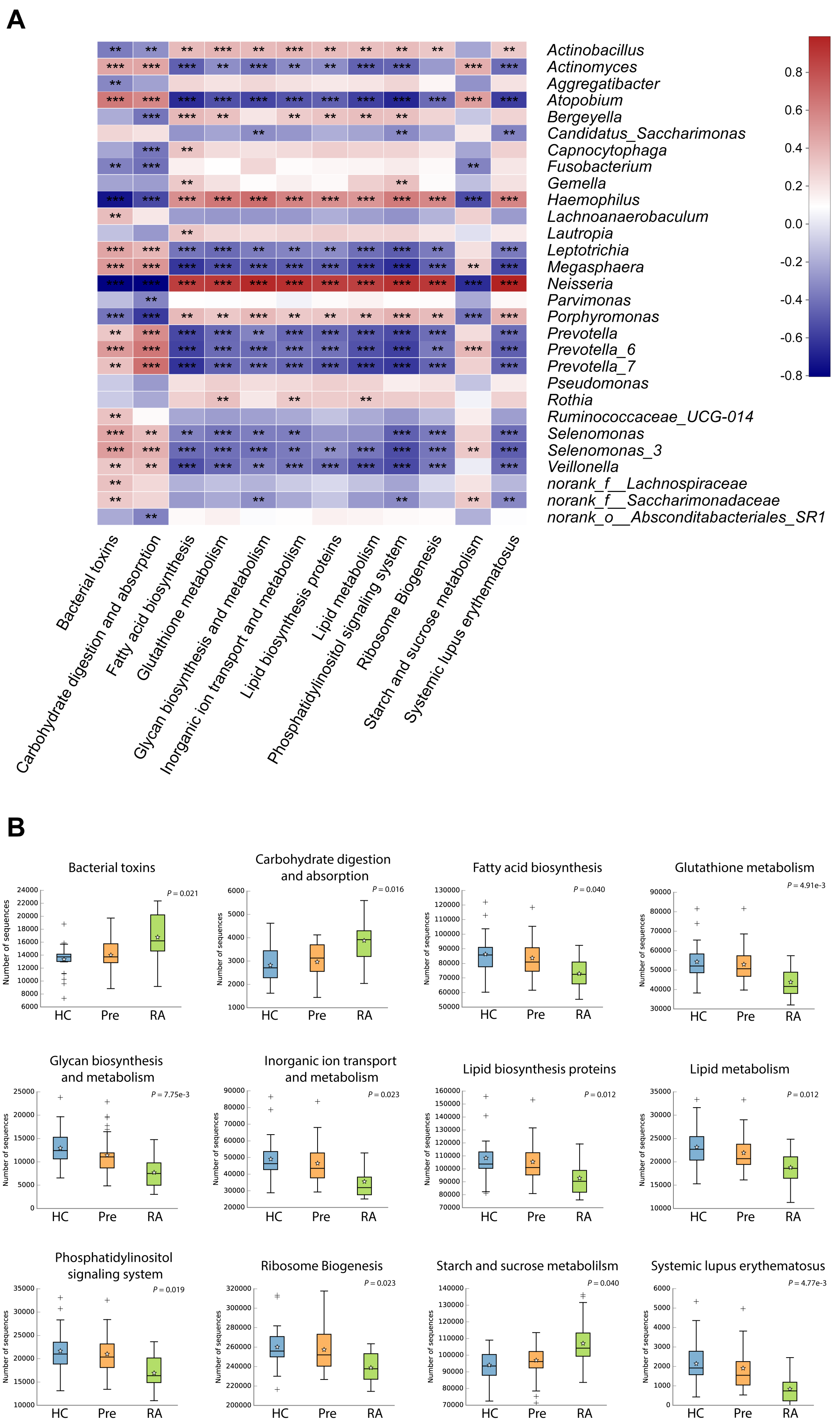
A
Actinobacillus
Actinomyces
Aggregatibacter
Atopobium
Bergeyella
Candidatus_Saccharimonas
Capnocytophaga
Fusobacterium
Gemella
Haemophilus
Lachnoanaerobaculum
Lautropia
Leptotrichia
Megasphaera
Neisseria
Parvimonas
Porphyromonas
Prevotella
Prevotella_6
Prevotella_7
Pseudomonas
Rothia
Ruminococcaceae_UCG-014
Selenomonas
Selenomonas_3
Veillonella
norank_f__Lachnospiraceae
norank_f__Saccharimonadaceae
norank_o__Absconditabacteriales_SR1
0.8
0.6
0.4
0.2
0.0
-0.2
-0.4
-0.6
-0.8
Bacterial toxins
Carbohydrate digestion and absorption
Fatty acid biosynthesis
Glutathione metabolism
Glycan biosynthesis and metabolism
Inorganic ion transport and metabolism
Lipid biosynthesis proteins
Lipid metabolism
Phosphatidylinositol signaling system
Ribosome Biogenesis
Starch and sucrose metabolism
Systemic lupus erythematosus
B
Bacterial toxins
P = 0.021
Carbohydrate digestion and absorption
P = 0.016
Fatty acid biosynthesis
P = 0.040
Glutathione metabolism
P = 4.91e-3
Glycan biosynthesis and metabolism
P = 7.75e-3
Inorganic ion transport and metabolism
P = 0.023
Lipid biosynthesis proteins
P = 0.012
Lipid metabolism
P = 0.012
Phosphatidylinositol signaling system
P = 0.019
Ribosome Biogenesis
P = 0.023
Starch and sucrose metabolilsm
P = 0.040
Systemic lupus erythematosus
P = 4.77e-3
Number of sequences
HC
Pre
RA

Supplementary Figure 4 Predicted saliva microbiota functions are perturbed in RA patients. PICRUSt was applied to infer the functional content of the microbiota. (A) The heat map shows the associations between differentially abundant taxa with predicted functional KEGG (Kyoto Encyclopedia of Genes and Genomes) pathways. The color scale is based on the value of Spearman correlation coefficient and represents the magnitude of correlation. Red scale indicates positive correlations; blue scale indicates negative correlations. (B) Predicted functional changes are indicated in samples from RA patients. Boxes indicate the interquartile range (IQR) (75th to 25th of the data). The median value is shown as a line within the box and the mean value as a star. Whiskers extend to the most extreme value within 1.5*IQR. Outliers are shown as crosses.
